# Genetic and Functional Evaluation of the Role of FOXO1 in Antituberculosis Drug-Induced Hepatotoxicity

**DOI:** 10.1155/2021/3185874

**Published:** 2021-06-19

**Authors:** Jingwei Zhang, Lin Jiao, Jiajia Song, Tao Wu, Hao Bai, Tangyuheng Liu, Zhenzhen Zhao, Xuejiao Hu, Binwu Ying

**Affiliations:** ^1^Department of Laboratory Medicine, Chengdu Second People's Hospital, Chengdu, Sichuan 610017, China; ^2^Department of Laboratory Medicine, West China Hospital, Sichuan University, Chengdu, Sichuan, China

## Abstract

**Background:**

The accumulation of the hepatotoxic substance protoporphyrin IX (PPIX) induced by aminolevulinate synthase 1 (ALAS1) activation is one of the important mechanisms of antituberculosis drug-induced hepatotoxicity (ATDH). Forkhead box protein O1 (FOXO1) may activate ALAS1 transcription. However, little is known about their roles in ATDH; we performed a study to determine the association between polymorphisms in the two genes and ATDH susceptibility. Then, we verified this possible association by cellular functional experiments.

**Materials and Methods:**

Tag single-nucleotide polymorphisms (TagSNPs) in the two genes were genotyped in 746 tuberculosis patients. The frequencies of the alleles, genotypes, genetic models, and haplotype distribution of the variants were compared between the case and control groups. L-02 cells and HepG2 cells were incubated with the indicated concentration of isoniazid (INH) and rifampicin (RIF) for the desired times, and then the expression levels of ALAS1 and FOXO1 mRNAs and proteins were detected. HepG2 cells were transiently transfected with FOXO1 siRNA to observe the effect of changes in the FOXO1 expression on the cell survival rate and ALAS1 expression.

**Results:**

The C allele at rs2755237 and the T allele at rs4435111 in the FOXO1 gene were associated with a decreased risk of ATDH. The expression of ALAS1 in both L-02 cells and HepG2 cells was increased by the coadministration of INH/RIF (600/200 *μ*M) for 24 h. Although FOXO1 expression was reduced slightly by the same treatment, its content in the nucleus was significantly increased. However, the cell survival rate and ALAS1 expression level were not significantly altered by the downregulation of FOXO1 in HepG2 cells.

**Conclusions:**

Variants of the rs4435111 and rs2755237 loci in the FOXO1 gene were associated with susceptibility to ATDH. Coadministration of INH/RIF promoted the transfer of FOXO1 from the cytoplasm to the nucleus, but the functional significance of its nuclear translocation requires further verification.

## 1. Introduction

The ancient infectious disease tuberculosis (TB) still seriously threatens human health. According to the 2020 Global Tuberculosis Annual Report, about 10.0 million people developed TB disease in 2019, and there were an estimated 1.2 million TB deaths among them. Tuberculosis patients in China accounted for about 8.4% of the whole world [1]. Isoniazid (INH) and rifampicin (RIF) are the main drugs recommended by the WHO as the first-line antituberculosis treatments. The efficiency of this protocol is high as 85%, but the adverse reactions caused by coadministration of INH and RIF cannot be ignored. Among these adverse reactions, antituberculosis drug-induced hepatotoxicity (ATDH) is the most common and serious, with an incidence of 5.0–28.0% and mortality of 0.042–0.07‰ [2–4]. No obvious dose dependence on the occurrence of ATDH has been reported, and the incidence, clinical symptoms, and severity are quite different between individuals [5]. Although researchers have reported many achievements in recent decades, the mechanism has not been completely elucidated. Due to the lack of specific symptoms and diagnostic biomarkers, the diagnosis of ATDH currently remains a challenging clinical problem.

Pharmacogenomics is an important tool to study the differences in drug metabolism and transport capabilities in different individuals caused by their genetic background. Pharmacogenomics studies have identified genetic mutations present in individuals as independent risk factors for ATDH by analysing the correlation between susceptible loci of target genes and phenotypes of the drug response [6, 7]. Single-nucleotide polymorphisms (SNPs), the main molecular markers in pharmacogenomics, have been proven to have potential and clinical application value as biomarkers of ATDH. For example, due to the association between the N-acetyltransferase (NAT2) gene “slow acetylation” phenotype and the increased blood concentration of INH, accompanied by an increased incidence of hepatotoxicity, the FDA has included the NAT2 gene phenotype in the INH drug label [6]. The PHARMGKB database also recommends important NAT2 genetic variants as clinical markers for predicting the risk of ATDH caused by INH in patients with tuberculosis [8]. Studies of genetic polymorphisms in key signalling pathways involved in the mechanism of ATDH will be helpful to screen specific targets as biomarkers for the diagnosis, treatment effect and prognosis.

In 2012, Li et al. found that the coadministration of INH/RIF leads to the accumulation of the endogenous hepatotoxic substance protoporphyrin IX (PPIX) in the liver of humanized pregnane X receptor- (PXR-) expressing mice. PPIX accumulation caused by PXR/ALAS1 axis activation induced by the coadministration of INH/RIF was proposed to be one of the important mechanisms of ATDH. It has milestone significance in the research history of the ATDH mechanism [9, 10]. Aminolevulinate synthase 1 (ALAS1) is the first rate-limiting enzyme in the haem biosynthesis pathway. Aminolevulinic acid (ALA), the product of ALAS1, is the precursor of PPIX. The rate of ALA synthesis is positively correlated with the rate of haem synthesis to ensure sufficient haem supplies for cytochrome enzyme biosynthesis [11]. Not only does the transcriptional activation of ALAS coordinate with the content of haem but the content of haem in the liver also negatively regulates the transcriptional activation of ALAS1. For example, the relative lack of haem caused by the increased demand for cytochrome enzyme synthesis will activate ALAS transcription; moreover, excessive haem directly reduces ALAS1 mRNA stability and inhibits ALAS1 mRNA transcription. The balance between ALAS1 transcription and the haem content ensures that the transcription of ALAS1 maintains a low baseline activation state to prevent potential damage caused by excessive free haem and haem precursor accumulation [12, 13].

PXR is an important transcription factor that induces ALAS1 transcription [9–11]. However, the transcriptional balance of the ALAS1 gene does not completely depend on PXR. Different external or internal stimuli induce the activation of the corresponding signalling pathways to coordinate with the PXR/ALAS1 axis, such as the hepatocyte nuclear factor 4 (HNF4) pathway, which is sensitive to a growth hormone pulse: the forkhead box protein O1 (FOXO1) pathway, which is sensitive to insulin; and the peroxisome proliferator-activated receptor-*γ* coactivator-1*α* (PGC-1*α*) pathway, which is sensitive to hyperglycaemia [12, 14]. Notably, a high-concentration glucose infusion effectively and quickly relieves the clinical symptoms of acute porphyrin attacks, suggesting that blood glucose or blood glucose-related hormone levels may convert endogenous metabolic signals to ALAS1 transcriptional regulatory signals through FOXO1 or PGC-1*α* to maintain the porphyrin balance [14, 15]. The promoter of the ALAS1 gene in mice and poultry contains a ALAS1 drug-responsive enhancer sequence (ADRES), which is a highly homologous and conserved DNA sequence with the insulin responsive element (IRE) region of the gluconeogenic gene. FOXO1 initiates the transcription of gluconeogenesis-related genes by binding to the IRE domain [16]. FOXO1 in the nucleus interacts with PGC-1*α* to form a heterodimer, which promotes ALAS1 transcription by binding to ADRES in a familiar manner. The expression level of this heterodimer is positively correlated with the ALAS1 mRNA expression level in mice, suggesting that FOXO1 participates in the induction of ALAS1 transcription regulation in the form of a FOXO1/PGC-1*α* heterodimer [14, 17]. The human ALAS1 gene promoter region also contains a similar domain with the potential capability to bind the FOXO1/PGC-1*α* heterodimer, although the mechanism is unclear [13, 15, 18–20]. However, researchers have not yet clearly determined whether and how FOXO1 transforms the stimulation of antituberculosis drugs into ALAS1 gene expression.

Studies on the association of PXR and PGC-1*α* gene polymorphisms with the risk of ATDH have been reported [21–23]. However, to the best of our knowledge, no genetic associations between FOXO1 and ALAS1 variants and susceptibility to ATDH have been reported yet. Therefore, considering the grim situation of tuberculosis in China, the aim of the present study was to explore the possible association between FOXO1 and ALAS1 gene polymorphisms and the risk of ATDH in the Han Chinese population and verify the association by performing cytofunctional experiments.

## 2. Materials and Methods

### 2.1. Patient Recruitment and Definition of Hepatotoxicity

Seven hundred forty-six patients with a confirmed tuberculosis diagnosis were included in the study, all of whom were recruited at West China Hospital between December 2014 and April 2018, according to our inclusion and exclusion criteria described in previous studies [21, 24]. Briefly, the inclusion criteria for the ATDH group were as follows: (a) normal serum alanine aminotransferase (ALT) (0–40 IU/L) and aspartate aminotransferase (AST) (0–40 IU/L) levels before treatment; (b) ALT and/or AST levels ≥3 × upper limit of normal (ULN) (120 IU/L) with hepatitis symptoms; (c) ALT and/or AST levels ≥5 × ULN (200 IU/L) with or without symptoms; (d) total bilirubin (TBIL) levels ≥1.5 × ULN (42 *μ*mol/L); (e) not taking other potential hepatotoxic drugs; and (f) no history of infection with hepatitis virus or human immunodeficiency virus [25, 26]. The inclusion criteria for the non-ATDH group were normal serum ALT, AST, and TBIL levels before and after treatment. The process of enrolment is depicted in Figure S1. Ethical approval for this study was obtained from the Institutional Review Board of the West China Hospital of Sichuan University. The definition of drug-induced hepatotoxicity used in this study was based on the National Institutes of Health and Common Toxicity Criteria for Adverse Events v5.0 (CTCAE v5.0) [24, 25, 27]. The definition of the severity of hepatotoxicity was based on the WHO Adverse Drug Reaction Terminology: mild liver injury, ALT level<5 × upper limit of normal (ULN) (200 IU/L); moderate liver injury, ALT levels greater than 5 × ULN but less than 10 × ULN; severe liver injury ALT levels ≥10 × ULN (400 IU/L) [27].

### 2.2. Candidate Single-Nucleotide Polymorphism Selection and Genotyping

Candidate SNPs were selected using the following strategies: (1) SNPs located within 2000 bp upstream and 300 bp downstream of the ALAS1 and FOXO1 genomic regions by searching the dbSNP database (http://www.ncbi.nlm.nih.gov/projects/SNP/) and 1000 Genomes Project (http://www.1000genomes.org/) [28]; (2) SNPs with a minor allele frequency (MAF) ≥0.02 and linkage disequilibrium (LD) *r*^2^ ≥ 0.8 among Southern Han Chinese (CHS) populations; (3) prioritizing the inclusion of SNPs that may be related to the risk of ATDH or have potential functional significance according to previous research [21, 29].

All the samples and data used in this study were obtained from the Bio-Bank of resources “Tuberculosis Research” in the Department of Laboratory Medicine, West China Hospital, Sichuan University, China, as mentioned previously [29]. Genomic DNA was extracted using a QIAamp® DNA Blood Mini Kit (Qiagen, Germany) according to the manufacturer's instructions. The SNP genotyping work was conducted using a custom-by-design 2 × 48-Plex SNP scan TM Kit (Cat#: G0104, Gene sky Biotechnologies Inc., Shanghai, China) as described previously [30]. Thirty samples were randomly selected for double-blind experiments to ensure the repeatability and stability of the genotyping results, and all the genotype calling success rates were greater than 99.0% [23].

### 2.3. Cell Culture and Chemicals

The HepG2 cells and L-02 cells used in this experiment were purchased from Kesimo Biotechnology Co. Ltd. (Wuhan, China) and Saier Biotechnology Co. Ltd. (Wuhan, China), respectively. INH and RIF were purchased from Sigma (St. Louis, MO). L-02 cells and HepG2 cells were cultured in Dulbecco's modified Eagle's medium (HyClone; Logan, UT) supplemented with 10% foetal bovine serum, 100 units/ml penicillin G, and 100 mg/ml streptomycin in a humidified atmosphere of 5% CO_2_ at 37°C. INH and RIF were dissolved in dimethyl sulfoxide (DMSO) at final concentrations of 30/10 *μ*M, 75/25 *μ*M, 150/50 *μ*M, 300/100 *μ*M, 600/200 *μ*M, 1200/400 *μ*M, and 2400/800 *μ*M [31].

### 2.4. MTT Assay for Cytotoxicity

L-02 and HepG2 cells (5 × 10^3^ cells/well) were seeded in 96-well plates and exposed to the desired concentration of INH/RFP or blank control for the indicated time points after reaching 60% confluence. Then, the cells were incubated with 3-(4, 5-dimethyl-thiazol-2-yl)-2, 5-diphenyltetrazolium bromide (MTT) (0.5 mg/mL) at 37°C for 4 h to form formazan crystals. After removing the supernatant, formazan crystals were dissolved in DMSO (150 ml/well). The optical density was measured at 490 nm. Cell viability was normalized as a percentage of the control. The experiment was independently performed in triplicate [31].

### 2.5. Quantitative Reverse Transcription Polymerase Chain Reaction (RT-PCR)

RNA was extracted from L-02 and HepG2 cells treated with the desired concentrations of INH/RIF or blank control for 24 h. RNA (1 *μ*g) was reverse-transcribed with a cDNA synthesis kit (Roche, Switzerland). PCR was performed using the cDNA templates in a 20 *μ*L final volume with SYBR Premix Ex Taq (Roche, Switzerland) in the Bio-Rad CXF real-time PCR detection system (Bio-Rad, USA) according to the manufacturer's instructions. Primer sequences for RT-PCR are listed in Table S1. Normalized values were used to calculate changes in expression between control and INH/RIF-treated cells using fold changes = 2^−ΔΔCt^ to calculate the relative content of the amplified product. All assays were performed in triplicate [32].

### 2.6. Western Blotting (WB)

Cells were lysed with RIPA lysis buffer (GIBCO BRL, America) containing 0.1 mol/L phenylmethylsulfonyl fluoride (PMSF). The total protein expression level was determined using a BCA protein assay. Proteins (500 *μ*g) in cell lysates were separated by SDS-PAGE using a 10% tricine gel, transferred to PVDF membranes for 1.5 h at 4°C, blocked with 5% skim milk in Tris-buffered saline containing 0.1% Tween 20, and subsequently immunoblotted using anti-ALAS1 (1 : 1000) and anti-FOXO1 (1 : 300) antibodies and horseradish peroxidase-conjugated secondary antibodies (Proteintech, America). The method for protein quantification was to calculate the ratio of the brightness value protein band in the sample to the brightness value of the corresponding internal reference (GAPDH) band. After adjusting the brightness value of the protein bands, the control group was used as the standard value [31].

### 2.7. Nuclear Protein Extraction and Preparation

The indicated concentrations of INH/RIF or control were added to L-02 or HepG2 cells. Cells were washed twice with PBS and scraped into 300 *μ*l of RIPA buffer. Cells were collected by centrifugation, resuspended in 400 *μ*l of cold hypotonic buffer and incubated on ice for 10 min. The nuclei were pelleted by centrifugation, resuspended in 200 *μ*l of lysis buffer, and incubated on ice for 20 min. The suspension was collected and stored at −80°C. Cold hypotonic buffer contained 1 mL of hypotonic buffer + 5 *μ*L of phosphatase inhibitor + 10 *μ*L of PMSF + 1 *μ*L of dithiothreitol (DTT) [33].

### 2.8. CCK-8 Assay of the Cell Survival Rate

Cells in logarithmic growth phase were trypsinized to prepare cell suspensions, inoculated in 96-well plates (10,000 cells per well with 100 *μ*l of medium), and incubated in an incubator with a humidified atmosphere of 5% CO_2_ at 37°C overnight. The marginal wells were filled with sterile PBS. The plating time, transfection time, and measurement time were 24 h, 48 h, and 96 h after inoculation, respectively. Ten microlitres of CCK-8 solution was added to each well and incubated for 1 h. The absorbance value was measured at 450 nm with a microplate reader. The results were calculated from six independent experiments.

### 2.9. RNA Interference and Transient Transfection

An siRNA with a scrambled sequence was used as a negative control (NC siRNA). HepG2 cells were seeded in 6-well plates and cultured until reaching 70% confluence, and transfection mixtures containing 100 pmol of the FOXO1 siRNA or NC siRNA were transfected into the cells by lipofection with Lipofectamine™ 2000 Transfection Reagent (Invitrogen, USA) based on a method reported previously [32]. Transfected cells were incubated for another 24 h and harvested to determine mRNA and protein levels. Small interfering RNA (siRNA) sequences targeting FOXO1 used in the present study are shown in Table S2.

### 2.10. Statistical Analysis

The independent sample *t*-test and the Mann–Whitney *U* test were applied to analyse continuous variables according to the normality of the data. The chi-square test was used to analyse categorical variables. One-way ANOVA was applied to compare the means of each group with the control group. The statistical analyses mentioned above were assessed using IBM SPSS Statistics software version 22.0. Hardy–Weinberg equilibrium (HWE) for SNPs and the associations between the SNPs and ATDH adjusted for age and sex were assessed using Plink software version 1.07. Linkage disequilibrium (LD) and haplotype analysis were assessed using Haplotype software version 4.2 [29]. The odds ratio (OR) with the corresponding 95% confidence interval (CI) was used as a measure of association, and two-sided *p* < 0.05 was considered significant [34].

## 3. Results

### 3.1. General Characteristics of the Study Subjects

Compared with patients in the non-ATDH group, fewer patients in the ATDH group had a fever. At the same time, patients in the ATDH group had higher basal levels of total bilirubin (TBIL), conjugated bilirubin (DBIL), aspartate transaminase (AST), ALT, alkaline phosphatase (ALP), and glutamyl transferase (GGT), and lower basal levels of uric acid (UA) (all *p* < 0.05), as described in our previous article [21]. The demographic and clinical features of the patients are described in Table S3.

### 3.2. SNP Alleles, Genotypes, Genetic Models, and Haplotype Analysis

In this study, three ALAS1 SNPs (rs353556, rs3852071, and rs352169) and five FOXO1 SNPs (rs2755237, rs2701891, rs3751436, rs4435111, and rs7325594) were selected and successfully genotyped in the 746 participants. None of the SNP genotype distributions deviated from Hardy–Weinberg equilibrium (HWE) (*p* > 0.05 for all loci). The basic features of candidate single-nucleotide polymorphisms in the FOXO1 and ALAS1 genes are depicted in Table S4.

Carriers with the C allele at the rs2755237 locus of the FOXO1 gene had a lower risk of ATDH than those with the A allele (OR = 0.631; 95% CI: 0.423–0.942, *p*=0.024) after adjusting for age and sex. Carriers with the T allele at the rs4435111 locus of the FOXO1 gene had a lower risk of ATDH than carriers with the C allele (OR = 0.572; 95% CI: 0.372–0.879, *p*=0.010) after adjusting for age and sex. The CC/CA/AA genotype distribution frequency of the rs2755237 locus showed a borderline significant difference between the ATDH and non-ATDH groups (3.41%/38.46%/58.12% vs. 6.40%/46.88%/46.72%, *p*=0.059). The TT/CT/CC genotype frequency of the rs4435111 locus was statistically significantly different between the two groups (1.69%/27.12%/70.33% vs. 4.61%/37.10%/58.28%, *p*=0.026). The other loci in the FOXO1 gene and all loci in the ALAS1 gene showed no significant differences between the two groups in either allele or genotype frequencies, all *p* > 0.05. The allele and genotype frequencies are depicted in Table 1.

The genetic models of the two genes were analysed using additive, dominant, and recessive models. The dominant model of the rs2755237 locus showed that patients with the CC + CA genotype had a 36.9% reduction in the risk of ATDH compared with patients with the AA genotype (OR = 0.631; 95% CI: 0.423–0.942, *p*=0.024). The dominant model of the rs4355111 locus showed that patients with the TT + CT genotype had a 42.8% reduction in the risk of ATDH than patients with the CC genotype (OR = 0.572; 95% CI: 0.372–0.879, *p*=0.010). The additive model of the two loci also showed a reduced risk of ATDH (OR = 0.658; 95% CI: 0.464–0.932, *p*=0.018 and OR = 0.591, 95% CI: 0.401–0.869, *p*=0.007, respectively). The results of the genetic model analysis of candidate SNPs are depicted in Table 2.

**Figure 1:**
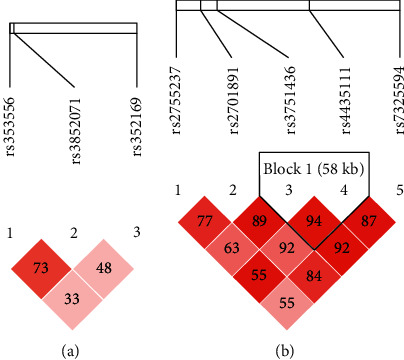
The haplotype analysis of the ALAS1 and FOXO1 genes. (a) The haplotype analysis of the ALAS1 gene. None of the SNPs of the ALAS1 gene showed tight linkage. (b) The haplotype analysis of the FOXO1 gene. rs3751436 and 4435111 are in the same linkage box (block 1) (dark red squares indicate that the two SNPs are tightly linked, and the numbers in the boxes represent D′ (unit %).

Linkage disequilibrium and haploid analyses can help to explore the combined effects of target SNPs on diseases of concern [35]. The linkage disequilibrium analysis showed that the rs3751436 and rs4435111 loci of the FOXO1 gene were in strong linkage disequilibrium (*D*′ = 0.92; *r*^2^ = 0.81), and the TT haplotype formed by the loci was less frequently distributed in the ATDH group than in the non-ATDH group (15.2% vs. 22.6%, *p*=0.011), suggesting that these loci may be related to ATDH susceptibility. None of the SNPs in the ALAS1 gene showed a tight linkage, which was not suitable for the haplotype analysis. The haploid analysis is shown in Figure 1, and the correlation between the haplotype distribution and risk of ATDH is shown in Table 3.

We searched for associations between target SNP loci and clinical features of ATDH. Carriers of the CC genotype at the rs2755237 locus had lower white blood cell counts and blood glucose levels and higher levels of ALT. TT genotype carriers at the rs4435111 locus had a higher erythrocyte sedimentation rate (ESR) and lower levels of Dbil than CC genotype carriers, as shown in Tables S5 and S6. However, no correlation was found between different genotypes at the two loci and the severity of hepatotoxicity, as shown in Table S7.

### 3.3. Cytotoxicity of INH/RIF Administration in L-02 and HepG2 Cells

We used the MTT assay to detect the viability of L-02 cells after exposure to different concentrations of the combination of INH/RIF at 24 h or 48 h to select the appropriate drug concentration and treatment time for subsequent experiments. The cell viability in the 150/50 *μ*M, 300/100 *μ*M, and 600/200 *μ*M groups was 79.45%, 72.93%, and 66.84%, respectively, after 24 h of incubation. The cell viability was even lower after 48 h of incubation. Based on this result, the viability of L-02 cells was reduced in a drug concentration- and time-dependent manner. The administration of INH/RIF (600/200 *μ*M) significantly reduced cell viability after an incubation for 24 h. We also performed the MTT assay to detect the viability of HepG2 cells and avoid selection bias associated with the use of a single cell line, and the results showed that the cell viability of the 600/200 *μ*M group was reduced to 60.89% after 24 h of incubation (data available if necessary). The results described above indicated that the cytotoxicity of INH/RIF toward the two cell lines was similar. Therefore, we chose 24 h as the suitable INH/RIF administration time point and 600/200 *μ*M as the suitable concentrations in subsequent experiments, unless indicated otherwise.

### 3.4. ALAS1 and FOXO1 mRNA and Protein Expression in Cells Treated with INH/RIF

**Figure 2:**
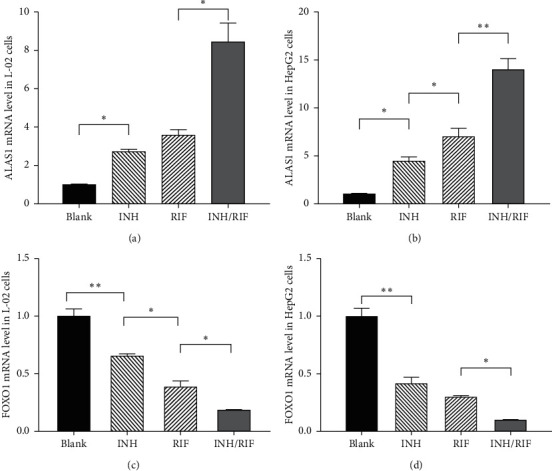
Effects of INH/RIF on the mRNA level of ALAS1 and FOXO1 in L-02 cells and HepG2 cells. (a) L-02 cells were treated without the drug as the blank control, with INH (600 *μ*M), RIF (200 *μ*M), or INH/RIF (600/200 *μ*M) for 24 h. ALAS1 mRNA expression levels were analysed by RT-PCR. (b) HepG2 cells were treated without the drug, with INH (600 *μ*M), RIF (200 *μ*M), or INH/RIF (600/200 *μ*M) for 24 h. ALAS1 mRNA expression levels were analysed by RT-PCR. (c) L-02 cells were treated with the same concentration of INH and/or RIF for 24 h. FOXO1 mRNA expression levels were analysed by RT-PCR. (d) HepG2 cells were treated with the same concentration of INH and/or RIF for 24 h. FOXO1 mRNA expression levels were analysed by RT-PCR.

**Figure 3:**
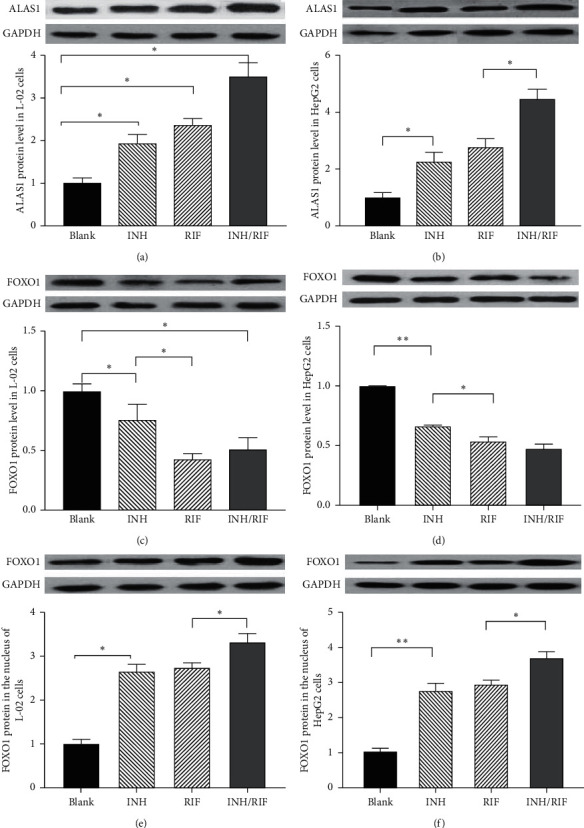
Effects of INH/RIF on the mRNA level of ALAS1 and FOXO1 in L-02 cells and HepG2 cells. (a) L-02 cells were treated without the drug as the blank control, with INH (600 *μ*M), RIF (200 *μ*M), or INH/RIF (600/200 *μ*M) for 24 h. ALAS1 protein expression levels were quantified by western blotting. (b) HepG2 cells were treated without the drug, with INH (600 *μ*M), RIF (200 *μ*M), or INH/RIF (600/200 *μ*M) for 24 h. ALAS1 protein expression levels were quantified by western blotting. (c) L-02 cells were treated with the same concentration of INH and/or RIF for 24 h. FOXO1 protein expression levels were quantified by western blotting. (d) HepG2 cells were treated with the same concentration of INH and/or RIF for 24 h. FOXO1 protein expression levels were quantified by western blotting. (e) L-02 cells were treated with the same concentration of INH and/or RIF for 24 h. Relative protein (in the nucleus) levels of FOXO1 were quantified by western blotting. (f) HepG2 cells were treated with the same concentration of INH and/or RIF for 24 h. Relative protein (in the nucleus) levels of FOXO1 were quantified by western blotting.

The ALAS1 mRNA level in the INH/RIF group of L-02 cells (8.521 ± 0.9036) was higher than that of the blank group (1.000 ± 0.01549), the INH group (2.762 ± 0.1005), and the RIF group (3.630–0.2291), all *p* < 0.0001. Thus, INH/RIF increased the expression of the ALAS1 mRNA in L-02 cells. Similarly, INH/RIF increased the expression level of the ALAS1 mRNA in HepG2 cells. The FOXO1 mRNA level in the INH/RIF group was slightly reduced compared with that of the blank group in both L-02 cells and HepG2 cells. The effects of INH/RIF on the levels of the ALAS1 and FOXO1 mRNAs in L-02 cells and HepG2 cells are depicted in Figure 2. Consistent with the mRNA data, INH/RFP increased the level of the ALAS1 protein and reduced the level of the FOXO1 protein after 24 h. The effects of INH/RIF on the levels of the ALAS1 and FOXO1 proteins in L-02 cells and HepG2 cells are depicted in Figure 3. As the transcription-promoting function of FOXO1 is correlated with its localization [14, 15], we also detected the FOXO1 protein content in the nucleus. Interestingly, in both L-02 and HepG2 cells, the level of FOXO1 in the nucleus increased significantly at 24 h after the administration of INH/RIF, as shown in Figure 3. Although INH/RIF slightly reduced the total amount of FOXO1 in the cells, it induced the transfer of FOXO1 from the cytoplasm to the nucleus through an as yet unknown mechanism.

### 3.5. Effect of FOXO1 on the Cell Survival Rate and ALAS Expression Level

**Figure 4:**
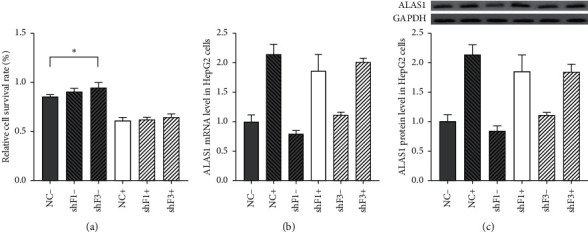
Effect of FOXO1 on cell survival rate and ALAS transcription level. (a) HepG2 cells were transiently transfected with siRNAs of FOXO1 (shF1 and shF3) or siRNA control. “−” = without coadministration of INH/RIF (600/200 *μ*M); “+” = with coadministration of INH/RIF (600/200 *μ*M) for 24 h. (b) HepG2 cells were transiently transfected with siRNAs of FOXO1 (shF1 and shF3) or siRNA control for 24 h. ALAS1 mRNA expression levels were quantified by RT-qPCR. (c) HepG2 cells were transiently transfected with siRNAs of FOXO1 (shF1 and shF3) or siRNA control for 24 h. ALAS1 protein expression levels were quantified by western blotting.

Since INH/RIF increased the level of the FOXO1 protein in the nucleus, we speculated that it might affect the cell survival rate by regulating ALAS1 transcription. Three siRNAs (FOXO1 shF1, FOXO1 shF2, and FOXO1 shF3) were designed to interfere with the expression level of FOXO1. FOXO1 mRNA levels were detected to determine the interference efficiency. The downregulation induced by shF1 and shF3 was stable and significant compared with that of the shF2 group (data available if necessary). Therefore, we chose shF1 and shF3 and transiently transfected HepG2 cells treated with or without INH/RIF to verify whether the expression of FOXO1 is correlated with the cell survival rate. The result of the CCK-8 assay is depicted in Figure 4. The cell survival rate of the shF3 group was slightly higher than that of the control group (0.9490 ± 0.0549 vs. 0.8559 ± 0.0175, *p*=0.0026) without INH/RIF. The cell survival rate appeared to be slightly higher than that of the control group treated with INH/RIF. Unfortunately, the difference was not significant (0.6410 ± 0.03846 vs. 0.6136 ± 0.02898, *p*=0.2443). The cell survival rates of the shF1 group and the control group were not significantly different after treatment with or without INH/RIF, all *p* > 0.05. The results indicated that FOXO1 may not exert a significant effect on INH/RIF-induced cytotoxicity.

FOXO1 regulates ALAS1 transcription through the PI3K/Akt signalling pathway along with insulin [15]. We transfected HepG2 cells with shF1, shF3, and siRNA controls to transfect HepG2 cells and detect ALAS1 mRNA and protein levels to clarify whether FOXO1 transformed the signal from INH/RIF stimulation to induce ALAS1 transcription. No difference in the expression level of the ALAS1 mRNA was observed between the two interference groups and the siRNA control group, regardless of treatment with INH/RIF. Consistent with the mRNA levels, the protein levels in the interference groups were also not significantly different from those in the control group, as depicted in Figure 4. Based on the results, FOXO1 exerted little effect on the expression level of ALAS1 following INH/RIF stimulation.

## 4. Discussion

This study analysed the correlation between polymorphisms in the FOXO1 and ALAS1 genes and susceptibility to ATDH. Variants in the rs4435111 and rs2755237 loci of the FOXO1 gene were associated with susceptibility to ATDH. The coadministration of INH/RIF to L-02 cells or HepG2 cells increased the expression of ALAS1 and reduced the expression of FOXO1, but these changes were accompanied by a relative increase in the FOXO1 level in the nucleus. We interfered with the FOXO1 expression level with siRNAs to clarify whether FOXO1 affects cell survival by inducing ALAS1 transcription and did not observe a significant change in the cell survival rate or ALAS1 expression level. Based on these results, FOXO1 might not be an important target in the ATDH mechanism, although it did have a certain correlation with the risk of ATDH.

The ALAS1 protein is the rate-limiting enzyme for haem synthesis in the liver by catalysing protoporphyrin production from ALA. Then, protoporphyrin is converted into ferrous haem through the action of ferrochelatase (FECH) protein, and finally, haem is produced. The imbalance in porphyrin metabolism caused by ALAS1 has been proven to be related to liver damage caused by carbamazepine, tetrachlorohydroquinone, and hexachlorobenzene [36–39]. We analysed three SNPs in the ALAS1 gene (rs353556, rs3852071, and rs352169) but did not observe correlations with the susceptibility to ATDH. The possible explanations for this result are described below. (1) One of the inclusion criteria for tagSNPs in this study was a MAF ≥ 0.02 in the Han population in southern China. It may exclude potentially related SNPs with a MAF < 0.02 (CHS). (2) Since the ALAS1 gene is genetically conserved, no genetic variant in ALAS1 has been definitely identified to be related to the clinical phenotype or severity of porphyria attack [14, 39]. (3) Porphyria balance is regulated by multiple factors, such as the internal environment and the body's physiological and pathological state, the external environment (such as drugs or pressure stimulation), and the cross-talk between the internal and the external environments. The occurrence of abnormal porphyrin metabolism is caused by multiple regulatory disorders at the same time, such as strong stimulation, elevated transcription levels, posttranscriptional modifications and inactivation of posttranslational negative feedback mechanisms, and other mechanisms, in the form of “multiple blows” [14].

FOXO1 gene is located on chromosome 13q14.11 and encodes the FOXO1 protein, which belongs to the FOXO family. FOXO1 could combine with a promoter by shuttling into the nucleus and activate the transcription activity of the target genes [40]. FOXO1 plays multiple important roles in regulating cell homeostasis, glucose metabolism, immune response, cell cycle progression, apoptosis, and aging [14, 15]. FOXO1 may regulate porphyrin metabolism by inducing the transcription of the ALAS1 gene following insulin stimulation, which may be an auxiliary mechanism of the PXR/ALAS1 axis [14]. The allele frequency, genotype frequency, genetic models, and haplotype frequency of the rs4533111 locus of the FOXO1 gene were significantly different between the ATDH group and the non-ATDH group. The allele frequency and genetic model of the rs2755237 locus of the FOXO1 gene were significantly different between the ATDH group and the non-ATDH group. Genome-wide expression quantitative trait loci (eQTL) data from multiple tissues in major Genotype-Tissue Expression (GTEx) project databases are widely used to annotate the biological functions of SNPs [41]. We retrieved the genomic eQTL data to predict the potential biological functions of rs4435111 and rs2755237 with HaploReg version 4.1 (https://pubs.broadinstitute.org/mammals/haploreg/haploreg.php) and found that rs4435111 is an eQTL for WBP4 gene expression in skeletal muscle tissue and skin, and rs2755237 is an eQTL for SLC25A19 gene expression in whole blood mononuclear cells. However, we have not yet determined whether these two loci are related to the expression of any genes in the liver. The biological function annotations are shown in Table S8. We also retrieved data from a website (http://bioinfo.bjmu.edu.cn/mirsnp/search/) to search for potential functional SNPs with strong linkage disequilibrium with rs2755237 or rs4533111, but did not obtain any valuable clues (data not shown). The location of SNPs is often related to the corresponding function: SNP mutations in the coding region may change the genetic code due to base changes or affect the translation kinetics due to the speed of ribosomes passing through a specific region of the mRNA that ultimately changes the structure of the encoded protein; SNPs in gene regulatory regions may regulate the function of genes by affecting the binding of transcriptional promoters or other transcription factors; and SNPs located in introns of genes may function due to variable splicing [42, 43]. Both rs4435111 and rs2755237 are located in the intron region of the FOXO1 gene. Functional experiments are needed to verify whether these SNPs affect gene transcription or translation through alternative splicing.

As a FOXO1 gene polymorphism was associated with ATDH susceptibility, we proposed that the expression of FOXO1 was involved in hepatotoxicity caused by INH/RIF by regulating ALAS1 transcription. L-02 cells and HepG2 cells were used to observe the effect of coadministration of INH/RIF on the expression of ALAS1 and FOXO1 and to verify this hypothesis. INH/RIF increased levels of the ALAS1 mRNA and protein, indicating that the porphyrin synthesis pathway was activated. Nevertheless, INH/RIF exerted a mild effect on the expression level of FOXO1, but these treatments significantly altered its cellular sublocalization, indicating that the FOXO1 protein was transferred from the cytoplasm to the nucleus through a certain unknown mechanism. Previous studies have confirmed that the intracellular localization and activation of FOXO1 under normal or hyperglycaemic conditions are mainly related to insulin levels. (1) Insulin reduces glucose levels by inhibiting gluconeogenesis though the PI3K/AKT/FOXO1 signalling pathway. Insulin induces FOXO1 phosphorylation at Thr-24, Ser-256, and Ser-322, and phosphorylated FOXO1 may be transferred from the nucleus to the cytoplasm and degraded by the ubiquitin-mediated proteasome pathway in the cytoplasm. With a reduced content of the FOXO1 protein in the nucleus, the transcriptional activation of gluconeogenesis-related genes in the liver is suppressed, resulting in reduced gluconeogenesis [14]. (2) Insulin may disrupt the formation of the FOXO1/PGC-1*α* heterodimer, which is necessary for the activation of ALAS1 gene transcription by binding to the drug response element in the ALAS1 promoter [15]. However, in the case of absolute or relatively insufficient insulin levels, such as fasting or infection stress, FOXO1 may be transferred from the cytoplasm and be retained in the nucleus under the action of the Jun-N-terminal kinase (JNK) signalling pathway [14]. RIF activates the JNK pathway through an as yet unknown mechanism [14, 44]. Therefore, we speculated that FOXO1 might be activated and transferred to the nucleus through the JNK pathway upon INH/RIF stimulation.

The JNK pathway plays an important role in cellular stress regulation, and thus, the translocation of FOXO1 stimulated by the JNK pathway may be consistent with the regulation of inflammation, which is somewhat different from our hypothesis. However, this finding was consistent with the results of the cell functional experiments. We found that the change in the FOXO1 expression level had no significant effect on the ALAS1 mRNA or protein level. In addition, it only exerted a slight effect on the cell survival rate. An increasing number of researchers have noticed that FOXO1 is not only an important regulatory gene for glucose and energy metabolism but also an important target for regulating inflammation [45, 46]. Brown et al. postulated that lipopolysaccharide (LPS) promoted increased production of tumor necrosis factor-*α* (TNF-*α*) and interleukin-6 (IL-6) through a FOXO1-mediated mechanism and suggested that FOXO1 was an activator of the inflammatory reaction. Fan et al. found that FOXO1 activates Toll-like receptor 4 (TLR4) and downstream inflammatory factors to initiate the inflammatory response, and activation of the FOXO1/TLR4 axis reversibly inactivates FOXO1 by phosphorylation to hinder the inflammation cascade [47]. Researchers speculated that FOXO1/TLR4 transform metabolic stress to an inflammatory response, with self-limiting negative feedback to avoid the excessive activation of inflammation [47]. FOXO1 may have a bidirectional regulatory role in inflammation. According to Jiang et al., the production of TNF-*α* and IL-6 induced by LPS through FOXO1/TLR4 depends on the JNK pathway, which further clarifies that the inflammatory regulation of FOXO1 is related to the JNK pathway [48]. According to the literature and our research results, we speculated that the changes in FOXO1 expression and cellular sublocalization might be secondary to the activation of the JNK pathway, and thus, the regulation of FOXO1 had no significant effect on cell survival or ALAS1 expression levels.

Our study has several strengths. (1) This study only included patients from West China Hospital, which is the medical centre providing the best quality medical care in Western China, to ensure the surveillance of ATDH with strict criteria to avoid misclassification. (2) The laboratory for testing was certified by the American Association of Pathologists (CAP) to ensure that all the laboratory data were of good quality and reliable. (3) Based on the analysis of the association between genetic polymorphisms and susceptibility to ATDH, we conducted cytofunctional tests to verify the possible correlation. Our research also has some limitations. (1) The rs4435111 and rs2755237 loci, which were found to be associated with susceptibility to ATDH, are both located in intron regions, and no functional SNPs with strong linkage disequilibrium with them were identified. Their functional significance in the ATDH mechanism was difficult to verify through cell-based experiments to some degree. (2) RNA interference did not definitely reduce the FOXO1 content in the nucleus. Although siRNAs effectively reduce the level of the total FOXO1 protein, they are not as effective as gene knockout. Due to the activation of the JNK pathway by INH/RIF or other mechanisms, FOXO1 was transferred from the cytoplasm to the nucleus. As the ALAS1 gene usually maintains a low baseline level of transcription [13], FOXO1 in the nucleus may be sufficiently effective to activate ALAS1 in collaboration with PXR. Therefore, more rigorous experimental designs are needed to clarify the effect of FOXO1 on ALAS1 transcription.

In conclusion, we found that genetic polymorphisms in rs4435111 and rs2755237 in FOXO1 were associated with susceptibility to ATDH. However, FOXO1 may not be an important target molecule in the ATDH mechanism, and the change in its expression and subcellular localization may be a form of secondary regulation mediated by the activation of the JNK pathway following INH/RIF stimulation to adapt to cell stress. The functional significance of the transfer of FOXO1 into the nucleus for ATDH still requires further verification.

## Figures and Tables

**Table 1:** The distributions of genotype and allele frequencies of all the selected SNPs.

Gene	dbSNP	Variant	Allele			*p*	Genotype		*p*
			ATDH	Non-ATDH	OR (95% CI)		ATDH	Non-ATDH	
			1/2	1/2			11/12/22	11/12/22	
ALAS1	rs353556	A > G	116/120	583/673	1.067 (0.683–1.666)	0.775	29/58/31	128/327/173	0.590
	rs3852071	C > T	30/204	210/1042	0.733 (0.466–1.154)	0.180	1/28/88	16/178/432	0.285
	rs352169	G > A	90/144	448/806	1.130 (0.752–1.697)	0.556	17/56/44	75/298/254	0.692
FOXO1	rs2755237	A > C	99/137	527/729	0.631 (0.423–0.942)	0.024	4/45/68	40/293/292	0.059
	rs2701891	T > C	53/181	373/877	1.176 (0.793–1.744)	0.418	13/44/61	42/236/350	0.244
	rs3751436	T > C	70/166	320/936	1.181 (0.775–1.798)	0.437	18/60/38	84/314/229	0.684
	rs4435111	C > T	96/136	482/772	0.572 (0.372–0.879)	0.010	2/32/83	29/233/366	0.026
	rs7325594	T > C	8/228	46/1210	1.432 (0.924–2.219)	0.107	18/68/32	82/327/218	0.266

*p*: the *p* value was calculated using a logistic regression method. “1” = mutant allele; “2” = wild-type allele; “11” = mutant homozygote; “12” = heterozygote; “22” = wild-type homozygote.

**Table 2:** Genetic models of SNPs related to ATDH in patients with tuberculosis.

Gene	dbSNP	Dominant model		Recessive model		Additive model	
		OR (95% CI)	*p*	OR (95% CI)	*p*	OR (95% CI)	*p*
ALAS1	rs353556	1.067 (0.683–1.666)	0.775	1.273 (0.802–2.020)	0.802	1.121 (0.843–1.488)	0.431
	rs3852071	0.733 (0.466–1.154)	0.180	0.328 (0.043–2.503)	0.043	0.724 (0.477–1.098)	0.128
	rs352169	1.130 (0.752–1.697)	0.556	1.251 (0.709–2.208)	0.709	1.129 (0.842–1.512)	0.417
FOXO1	rs2755237	0.631 (0.423–0.942)	0.024	0.517 (0.181–1.476)	0.181	0.658 (0.464–0.932)	0.018
	rs2701891	1.176 (0.793–1.744)	0.418	1.727 (0.896–3.328)	0.896	1.226 (0.906–1.660)	0.186
	rs3751436	1.181 (0.775–1.798)	0.437	1.187 (0.683–2.063)	0.683	1.139 (0.849–1.529)	0.384
	rs4435111	0.572 (0.372–0.879)	0.010	0.359 (0.084–1.526)	0.084	0.591 (0.401–0.869)	0.007
	rs7325594	1.432 (0.924–2.219)	0.107	1.196 (0.688–2.080)	0.688	1.255 (0.931–1.691)	0.135

*p*: the *p* value was calculated using the logistic regression method. “1” = mutant allele; “2” = wild-type allele; “11” = mutant homozygote; “12” = heterozygote; “22” = wild-type homozygote.

**Table 3:** Associations of haplotypes constructed with variants of FOXO1 and the risk of ATDH.

Gene	Related loci	Haplotype	Frequency of the haplotype^*∗*^		*p*
			ATDH	Non-ATDH	
FOXO1	rs3751436 : rs4435111	T : C	0.434	0.390	0.2057
	rs3751436 : rs4435111	C : C	0.412	0.379	0.3360
	rs3751436 : rs4435111	T : T	0.152	0.226	0.0111

^*∗*^The ratio is shown according to the CC frequency.

## Data Availability

The data used to support the findings of this study are included within the article and the supplementary information file.

## Abbreviations

TB: Tuberculosis
RIF: Rifampicin
INH: Isoniazid
ATDH: Antituberculosis drug-induced hepatotoxicity
SNPs: Single-nucleotide polymorphisms
NAT2: N-Acetyltransferase
PPIX: Protoporphyrin IX
PXR: Pregnane X receptor
ALAS1: Aminolevulinate synthase 1
ALA: Aminolevulinic acid
HNF4: Hepatocyte nuclear factor 4
FOXO1: Forkhead box protein O1
PGC-1*α*: Peroxisome proliferator-activated receptor-*γ* coactivator-1*α*
ADRES: ALAS1 drug-responsive enhancer sequence
IRE: Insulin responsive element
CTCAE v5.0: National Institutes of Health and Common Toxicity Criteria for Adverse Events v5.0
ALT: Alanine aminotransferase
ULN: Upper limit of normal
MAF: Minor allele frequencies
LD: Linkage disequilibrium
CHS: Southern Han Chinese
MTT: 3-(4, 5-Dimethylthiazol-2-yl)-2, 5-diphenyltetrazolium bromide
DMSO: Dimethyl sulfoxide
RT-PCR: Quantitative reverse-transcriptase polymerase chain reaction
WB: Western blotting
PMSF: Phenylmethylsulfonyl fluoride
DTT: Dithiothreitol
TBIL: Total bilirubin
DBIL: Conjugated bilirubin
AST: Aspartate transaminase
ALP: Alkaline phosphatase
GGT: Glutamyl transferase
UA: Uric acid
HWE: Hardy–Weinberg equilibrium
ESR: Erythrocyte sedimentation rate
siRNA: Small interfering RNA
FECH: Ferrochelatase
eQTL: Genome-wide expression quantitative trait loci
GTEx: Genotype-tissue expression
JNK: Jun-N-terminal kinase
LPS: Lipopolysaccharide
TNF-*α*: Tumor necrosis factor-*α*
IL-6: Interleukin-6
TLR4: Toll-like receptor 4
CAP: American Association of Pathologists.

## Supplementary Materials

Supplementary Materials Figure S1: flow diagram of the study population. Table S1: primer sequences for RT-PCR. Table S2: siRNA sequences targeting FOXO1 used in the study. Table S3: demographic and clinical characteristics and laboratory indicators of enrolled patients. Table S4: candidate single-nucleotide polymorphism of FOXO1 and ALAS1. Table S5: correlation between laboratory indicators and the genotype of the rs2755237 locus. Table S6: correlation between laboratory indicators and the genotype of the rs4435111 locus. Table S7: analysis of the association of genotype distribution and different grades of severity. Table S8: potential biological function annotation for the SNPs related to ATDH.
