# Supplementary material for: Genetic and Functional Evaluation of the Role of FOXO1 in Antituberculosis Drug-Induced Hepatotoxicity
Source: Evid Based Complement Alternat Med. 2021 Jun 19;2021:3185874. doi: 10.1155/2021/3185874 (PMC8238576; doi:10.1155/2021/3185874)
Supplement: Supplementary Materials — Figure S1: flow diagram of the study population. Table S1: primer sequences for RT-PCR. Table S2: siRNA sequences targeting FOXO1 used in the study. Table S3: demographic and clinical characteristics and laboratory indicators of enrolled patients. Table S4: candidate single-nucleotide polymorphism of FOXO1 and ALAS1. Table S5: correlation between laboratory indicators and the genotype of the rs2755237 locus. Table S6: correlation between laboratory indicators and the genotype of the rs4435111 locus. Table S7: analysis of the association of genotype distribution and different grades of severity. Table S8: potential biological function annotation for the SNPs related to ATDH. [file 3185874.f1.zip › 3185874.f1/S2 Table siRNA.docx]

| NC batch | Cat# siN0000001-1-5 |
| --- | --- |
| FOXO1 siRNA sequence 1 | GAGGTATGAGTCAGTATAA |
| FOXO1 siRNA sequence 2 | GCCCTCGAACTAGCTCAAA |
| FOXO1 siRNA sequence 3 | TTCGGAATGACCTCATGGA |

S2 Table. siRNA sequences targeting FOXO1 used in the study.
