# Supplementary material for: Genetic and Functional Evaluation of the Role of FOXO1 in Antituberculosis Drug-Induced Hepatotoxicity
Source: Evid Based Complement Alternat Med. 2021 Jun 19;2021:3185874. doi: 10.1155/2021/3185874 (PMC8238576; doi:10.1155/2021/3185874)
Supplement: Supplementary Materials — Figure S1: flow diagram of the study population. Table S1: primer sequences for RT-PCR. Table S2: siRNA sequences targeting FOXO1 used in the study. Table S3: demographic and clinical characteristics and laboratory indicators of enrolled patients. Table S4: candidate single-nucleotide polymorphism of FOXO1 and ALAS1. Table S5: correlation between laboratory indicators and the genotype of the rs2755237 locus. Table S6: correlation between laboratory indicators and the genotype of the rs4435111 locus. Table S7: analysis of the association of genotype distribution and different grades of severity. Table S8: potential biological function annotation for the SNPs related to ATDH. [file 3185874.f1.zip › 3185874.f1/S3 Table. Demographic.docx]

| S3 Table. Demographic, clinical characteristics and laboratory indicators of enrolled patients. | | | | | | | | |
| --- | --- | --- | --- | --- | --- | --- | --- | --- |
| group | Non-ATDH(n=628) | | | | ATDH(n=118) | | p value | |
| General data | (n, %) | | | | | | | |
| Age (years）^a^ | 42.85±18.44 | | | 40.92±15.72 | | | | 0.284 |
| gender(male/female) ^c^ | 375(59.71%) | 253(40.28%) | | 69(58.47%) | | 49(41.52%) | | 0.801 |
| Smoking (No/Yes) ^c^ | 407(64.80%) | 221(35.19%) | | 80(67.79%) | | 38(32.20%) | | 0.532 |
| Drinking (No/Yes) ^c^ | 465(74.04%) | 163(25.95%) | | 83(70.33%) | | 35(29.66%) | | 0.464 |
| General symptoms (No/Yes) ^c^ | 135（19.62%） | 492(80.37%） | | 23(19.49%) | | 95(80.51%） | | 0.567 |
| Fever (No/Yes) ^c^ | 344（54.78%） | 284(45.22%） | | 50(42.37%) | | 68(57.62%） | | ***0.016*** |
| Weight loss (No/Yes) ^c^ | 367(58.43%) | 261(41.56%) | | 82（69.49%） | | 36(30.50%） | | 0.136 |
| Night sweat (No/Yes) ^c^ | 433(68.94%) | 195(31.05%) | | 86（72.88%） | | 32(27.12%） | | 0.446 |
| Fatigue (No/Yes) ^c^ | 462(73.57%) | 166(26.43%) | | 85(72.03%) | | 33(27.97%) | | 0.716 |
| Poor appetite (No/Yes) ^c^ | 374(59.55%) | 254(40.45%) | | 69（58.47%） | | 49(41.52%) | | 0.859 |
| Local infection (No/Yes) ^c^ | 134(21.34%) | 494(78.66%) | | 24（20.34） | | 94(79.66%) | | 0.758 |
| Laboratory indicators | Mean ± SD or P_50_(P_25_-P_75_) | | | | | | | |
| RBC (×10^12^/L) ^a^ | 4.28 ± 0.68 | | 4.31 ± 0.74 | | | | | 0.481 |
| HB(g/L) ^a^ | 122.06 ± 20.58 | | 122.87 ± 22.11 | | | | | 0.717 |
| HCT(L/L) ^a^ | 0.36 ± 0.06 | | 0.38 ± 0.06 | | | | | 0.069 |
| PLT (×10^9^/L) ^b^ | 232.50(172.75-297.25) | | 236.50(184.00-321.75) | | | | | 0.134 |
| WBC (×10^9^/L) ^b^ | 6.51(5.17-8.44) | | 6.57(4.99-7.96) | | | | | 0.761 |
| Neutrophil (×10^9^/L) ^a^ | 5.10 ± 2.73 | | 5.23 ± 2.89 | | | | | 0.631 |
| Monocyte (×10^9^/L) ^a^ | 1.26 ± 0.62 | | 1.29 ± 0.79 | | | | | 0.625 |
| Lymphocyte (×10^9^/L) ^a^ | 0.50 ± 0.25 | | 0.55 ± 0.29 | | | | | 0.099 |
| Neutrophil (%) ^a^ | 70.13 ± 11.54 | | 70.49 ± 11.50 | | | | | 0.760 |
| Monocyte (%) ^a^ | 7.30 ± 2.37 | | 7.74 ± 2.62 | | | | | 0.077 |
| Lymphocyte (%) ^b^ | 17.5(12.18-25.68) | | 16.25(12.58-25.58) | | | | | 0.527 |
| TBIL (μmol/L) ^b^ | 8.70(6.30-12.10) | | 10.05(7.50-14.13) | | | | | ***0.002*** |
| DBIL (umol/L) ^b^ | 3.45(2.50-5.40) | | 3.55(2.38-5.60) | | | | | 0.126 |
| IBIL (umol/L) | 4.80(3.40-7.03) | | 5.70(3.98-7.95) | | | | | ***0.049*** |
| ALT (IU/L) ^b^ | 15.00(10.00-21.00) | | 28.00(15.75-38.00) | | | | | ***<0.001*** |
| AST (IU/L) | 19.50(16.00-25.00) | | 27.00(20.00-34.00) | | | | | ***<0.001*** |
| TP(g/L) ^a^ | 68.82 ± 9.15 | | 69.42 ± 8.42 | | | | | 0.508 |
| ALB(g/L) ^a^ | 37.89 ± 6.90 | | 38.64 ± 7.35 | | | | | 0.248 |
| GLB(g/L) ^a^ | 30.93 ± 7.02 | | 30.78 ± 6.65 | | | | | 0.829 |
| GLU (mmol/L) ^b^ | 5.14(4.71-5.89) | | 5.15(4.64-5.95) | | | | | 0.41 |
| UREA (mmol/L) ^b^ | 4.05(3.15-5.30) | | 3.92(2.90-5.24) | | | | | 0.299 |
| CREA(μmol/L) ^b^ | 60.45(49.00-73.20) | | 57.50(47.78-67.00) | | | | | 0.601 |
| CYS-C(mg/L) ^b^ | 0.92(0.79-1.06) | | 0.91(0.81-1.04) | | | | | 0.975 |
| Uric(umol/L) ^a^ | 331.51 ± 155.30 | | 291.29 ± 125.98 | | | | | ***0.008*** |
| TG (mmol/L) ^b^ | 1.06(0.80-1.43) | | 0.99(0.81-1.31) | | | | | 0.469 |
| CHOL(mmol/L) ^a^ | 3.96 ± 1.058 | | 3.96 ± 1.206 | | | | | 0.966 |
| HDL-C(mmol/L) ^a^ | 1.08(0.82-1.41) | | 1.12(0.85-1.48) | | | | | 0.811 |
| LDL-C(mmol/L) ^b^ | 2.21(1.69-2.77) | | 2.20(1.79-2.72) | | | | | 0.575 |
| ALP(IU/L) ^b^ | 79.00(64.00-98.00) | | 85.50(68.50-106.00) | | | | | ***0.021*** |
| GGT(IU/L) ^b^ | 29.00(19.00-48.00) | | 42.50(26.00-78.00) | | | | | ***<0.001*** |
| CRP (mg/L) ^b^ | 12.25(2.67-37.43) | | 9.74(2.30-39.23) | | | | | 0.961 |
| ESR (mm/h) ^b^ | 33.50(14.75-64.00) | | 38.50(20.50-63.00) | | | | | 0.173 |

TB. tuberculosis; PTB, pulmonary tuberculosis; EPTB, extra-pulmonary tuberculosis.

^a^ Data shown as mean ± standard deviation; ^b^data shown as median, interquartile range; ^c^ data shown as number of cases (frequency)
