# Supplementary material for: Genetic and Functional Evaluation of the Role of FOXO1 in Antituberculosis Drug-Induced Hepatotoxicity
Source: Evid Based Complement Alternat Med. 2021 Jun 19;2021:3185874. doi: 10.1155/2021/3185874 (PMC8238576; doi:10.1155/2021/3185874)
Supplement: Supplementary Materials — Figure S1: flow diagram of the study population. Table S1: primer sequences for RT-PCR. Table S2: siRNA sequences targeting FOXO1 used in the study. Table S3: demographic and clinical characteristics and laboratory indicators of enrolled patients. Table S4: candidate single-nucleotide polymorphism of FOXO1 and ALAS1. Table S5: correlation between laboratory indicators and the genotype of the rs2755237 locus. Table S6: correlation between laboratory indicators and the genotype of the rs4435111 locus. Table S7: analysis of the association of genotype distribution and different grades of severity. Table S8: potential biological function annotation for the SNPs related to ATDH. [file 3185874.f1.zip › 3185874.f1/S4 Table tagSNPs.docx]

S4 Table. Candidate single nucleotide polymorphism of FOXO1 and ALAS1.

| Gene | dbSNP | allele | Position  (GRCh38.p7) | HWE | MAF | MAF* | |
| --- | --- | --- | --- | --- | --- | --- | --- |
| ALAS1 | rs353556 | A>G | chr3:52177806 | 0.855 | G=0.493 | | 0.468 |
|  | rs3852071 | C>T | chr3:52178708 | 0.689 | T=0.181 | | 0.161 |
|  | rs352169 | G>A | chr3:52202746 | 1.000 | A=0.395 | | 0.361 |
| FOXO1 | rs2755237 | A>C | chr13:40535292 | 0.428 | C=0.337 | | 0.287 |
|  | rs2701891 | T>C | chr13:40550515 | 0.270 | C=0.227 | | 0.261 |
|  | rs3751436 | T>C | chr13:40560901 | 0.567 | C=0.402 | | 0.389 |
|  | rs4435111 | C>T | chr13:40619338 | 0.346 | T=0.233 | | 0.219 |
|  | rs7325594 | T>C | chr13:40675585 | 1.000 | C=0.406 | | 0.399 |

MAF: minor allele frequency in 1000 Genomes (East Asia) <https://www.ncbi.nlm.nih.gov/snp>.

*: MAF Calculated by Haploview software in our study
