# Supplementary material for: Genetic and Functional Evaluation of the Role of FOXO1 in Antituberculosis Drug-Induced Hepatotoxicity
Source: Evid Based Complement Alternat Med. 2021 Jun 19;2021:3185874. doi: 10.1155/2021/3185874 (PMC8238576; doi:10.1155/2021/3185874)
Supplement: Supplementary Materials — Figure S1: flow diagram of the study population. Table S1: primer sequences for RT-PCR. Table S2: siRNA sequences targeting FOXO1 used in the study. Table S3: demographic and clinical characteristics and laboratory indicators of enrolled patients. Table S4: candidate single-nucleotide polymorphism of FOXO1 and ALAS1. Table S5: correlation between laboratory indicators and the genotype of the rs2755237 locus. Table S6: correlation between laboratory indicators and the genotype of the rs4435111 locus. Table S7: analysis of the association of genotype distribution and different grades of severity. Table S8: potential biological function annotation for the SNPs related to ATDH. [file 3185874.f1.zip › 3185874.f1/S5 Table clinic characteristic2755237.docx]

S5 Table. Correlation between laboratory indicators and the genotype of rs2755237 locus.

| Laboratory indicaters | rs2755237 | | | | *p* |
| --- | --- | --- | --- | --- | --- |
|  | AA | AC | CC |  |  |
| RBC (×10^12^/L) ^a^ | 4.26（3.97-4.74） | 4.51（4.11-4.93） | 4.13（4.08-4.27） | | 0.100 |
| HB (g/L) ^a^ | 121.00±24.60 | 124.00±18.70 | 126.00±9.45 | | 0.802 |
| HCT (L/L) ^a^ | 0.37±0.07 | 0.38±0.05 | 0.36±0.02 |  | 0.644 |
| PLT (×10^9^/L) ^b^ | 203（164-302） | 254（209-369） | 243（199-265.） | | 0.076 |
| WBC (×10^9^/L) ^b^ | 6.82（5.05-8.83） | 6.76（5.75-8.46） | 4.42（3.81-5.32） | | 0.025 |
| Neutrophil (%) ^a^ | 73.00（66.00-79.00） | 68.00（59.20-77.70） | 69.00（63.05-69.70） | | 0.075 |
| Monocyte (%) ^a^ | 18.20（12.37-26.47） | 16.30（13.80-23.20） | 25.00（15.00-35.00） | | 0.157 |
| Lymphocyte (%) ^b^ | 7.55±2.51 | 7.97±2.77 | 8.64±2.90 |  | 0.569 |
| Neutrophil (×10^9^/L) ^a^ | 4.83（3.31-6.54） | 4.43（3.27-6.31） | 3.07（2.48-3.67） | | 0.056 |
| Monocyte (×10^9^/L) ^a^ | 1.20±0.58 | 1.42±1.03 | 1.11±0.37 |  | 0.301 |
| Lymphocyte (×10^9^/L) ^a^ | 0.53±0.27 | 0.58±0.32 | 0.41±0.17 |  | 0.448 |
| CRP (mg/L) ^b^ | 11.80（4.32-36.45） | 13.40（2.02-43.20） | 1.79（1.27-10.10） | | 0.056 |
| ESR (mm/h) ^b^ | 45.50（19.50-79.00） | 35.00（18.00-61.00） | 54.00（15.00-84.00） | | 0.496 |
| TBIL (μmol/L) ^b^ | 10.50（7.05-13.90） | 10.30（7.52-15.20） | 11.95（5.00-20.4） | | 0.821 |
| DBIL (umol/L) ^b^ | 3.60（2.30-6.80） | 3.70（2.80-7.90） | 2.20（2.10-5.60） | | 0.195 |
| IBIL (umol/L) | 6.20（4.40-8.20） | 4.65（3.30-6.85） | 8.40（2.90-14.80） | | 0.301 |
| ALT (IU/L) ^b^ | 25.00（13.00-38.00） | 29.00（17.25-39.50） | 23.50（12.00-33.00） | | 0.314 |
| AST (IU/L) | 24.80±8.03 | 28.70±8.02 | 30.00±8.75 | | 0.030 |
| TP (g/L) ^a^ | 68.80±7.80 | 70.00±9.26 | 72.50±10.7 | | 0.556 |
| ALB (g/L) ^a^ | 38.20±7.23 | 39.10±7.67 | 39.60±8.02 | | 0.790 |
| GLB (g/L) ^a^ | 30.50±7.07 | 30.80±5.85 | 32.90±9.64 | | 0.767 |
| GLU (mmol/L) ^b^ | 5.21（4.73-5.97） | 4.88（4.50-5.85） | 4.55（4.25-4.83） | | 0.034 |
| UREA (mmol/L) ^b^ | 4.03（2.90-5.31） | 3.99（3.00-4.9） | 2.90（2.45-6.17） | | 0.073 |
| CREA (μmol/L) ^b^ | 61.00（53.00-73.00） | 60.10（48.00-71.20） | 51.20（44.00-85.00） | | 0.614 |
| CYS-C (mg/L) ^b^ | 0.98±0.42 | 1.07±0.55 | 0.85±0.14 |  | 0.485 |
| Uric(umol/L) ^a^ | 292.00±139.00 | 286.00±108.00 | 346.00±77.70 | | 0.664 |
| TG (mmol/L) ^b^ | 0.97（0.82-1.47） | 1.01（0.81-1.24） | 0.94（0.86-1.08） | | 0.182 |
| CHOL (mmol/L) ^a^ | 3.89（3.12-4.52） | 3.63（3.09-4.53） | 3.98（3.61-4.39） | | 0.825 |
| HDL-C (mmol/L) ^a^ | 1.20±0.48 | 1.10±0.47 | 1.52±0.22 |  | 0.182 |
| LDL-C (mmol/L) ^b^ | 2.19（1.68-2.63） | 2.15（1.65-2.73） | 1.95（1.77-2.21） | | 0.805 |
| ALP (IU/L) ^b^ | 103.00±85.90 | 92.00±32.10 | 84.50±20.30 | | 0.653 |
| GGT (IU/L) ^b^ | 39.00（25.00-72.00） | 47.50（28.00-82.75） | 37.00（30.75-53.75） | | 0.724 |

^a^ Data shown as mean ± standard deviation; ^b^data shown as median, interquartile range; ^c^ data shown as number of cases (frequency)
