# Supplementary material for: Genetic and Functional Evaluation of the Role of FOXO1 in Antituberculosis Drug-Induced Hepatotoxicity
Source: Evid Based Complement Alternat Med. 2021 Jun 19;2021:3185874. doi: 10.1155/2021/3185874 (PMC8238576; doi:10.1155/2021/3185874)
Supplement: Supplementary Materials — Figure S1: flow diagram of the study population. Table S1: primer sequences for RT-PCR. Table S2: siRNA sequences targeting FOXO1 used in the study. Table S3: demographic and clinical characteristics and laboratory indicators of enrolled patients. Table S4: candidate single-nucleotide polymorphism of FOXO1 and ALAS1. Table S5: correlation between laboratory indicators and the genotype of the rs2755237 locus. Table S6: correlation between laboratory indicators and the genotype of the rs4435111 locus. Table S7: analysis of the association of genotype distribution and different grades of severity. Table S8: potential biological function annotation for the SNPs related to ATDH. [file 3185874.f1.zip › 3185874.f1/S6 Table clinic character rs4435111 locus.docx]

S6 Table. Correlation between laboratory indicators and the genotype of rs4435111 locus.

| Laboratory indicators | rs4435111 | | | *p* |
| --- | --- | --- | --- | --- |
|  | CC | CT | TT |  |
| RBC (×10^12^/L) ^a^ | 4.28（3.98-4.75） | 4.58（4.13-4.93） | 3.74±0.64 | 0.103 |
| HB (g/L) ^a^ | 120.00±22.60 | 129.00±19.600 | 107.00±14.80 | 0.092 |
| HCT (L/L) ^a^ | 0.36±0.06 | 0.39±0.05 | 0.34±0.03 | 0.113 |
| PLT (×10^9^/L) ^b^ | 235（169-312） | 243（199-346） | 301.00±121.00 | 0.280 |
| WBC (×10^9^/L) ^b^ | 6.62（5.15-8.26） | 6.76（5.16-9.455） | 6.74±1.33 | 0.506 |
| Neutrophil (%) ^a^ | 73.00（62.60-79.40） | 69.00（64.30-74.65） | 67.30±14.70 | 0.792 |
| Monocyte (%) ^a^ | 15.20（10.30-27.38） | 15.80（12.20-26.10） | 18.89±10.49 | 0.202 |
| Lymphocyte (%) ^b^ | 7.58±2.52 | 8.07±2.87 | 9.85±1.20 | 0.354 |
| Neutrophil (×10^9^/L) ^a^ | 4.43（3.30-6.31） | 5.05（3.31-7.50） | 4.64±1.89 | 0.136 |
| Monocyte (×10^9^/L) ^a^ | 1.23±0.63 | 1.41±1.11 | 1.32±0.79 | 0.202 |
| Lymphocyte (×10^9^/L) ^a^ | 0.52±0.25 | 0.59±0.36 | 0.67±0.21 | 0.459 |
| CRP (mg/L) ^b^ | 12.90（3.60-36.32） | 5.71（2.02-43.20） | 21.00±28.30 | 0.271 |
| ESR (mm/h) ^b^ | 47.80±32.00 | 38.40±31.10 | 62.00±57.90 | 0.048 |
| TBIL (μmol/L) ^b^ | 10.90（7.60-14.60） | 9.95（7.50-17.45） | 5.35±0.77 | 0.788 |
| DBIL (umol/L) ^b^ | 3.60（2.80-6.75） | 3.95（2.30-6.95） | 1.95±0.49 | 0.048 |
| IBIL (umol/L) | 6.00（4.15-8.20） | 5.50（3.30-7.02） | 3.4±1.27 | 0.508 |
| ALT (IU/L) ^b^ | 27.00（13.50-38.00） | 31.50（20.00-41.50） | 24.50±12.00 | 0.049 |
| AST (IU/L) | 25.50±8.42 | 28.70±7.32 | 29.00±11.30 | 0.166 |
| TP (g/L) ^a^ | 68.50±8.38 | 71.30±8.46 | 73.50±9.61 | 0.227 |
| ALB (g/L) ^a^ | 37.90±7.61 | 40.50±6.67 | 37.60±5.09 | 0.252 |
| GLB (g/L) ^a^ | 30.50±6.60 | 30.80±6.97 | 35.90±4.52 | 0.541 |
| GLU (mmol/L) ^b^ | 5.08（4.59-5.78） | 4.87（4.53-5.92） | 5.65±1.07 | 0.419 |
| UREA (mmol/L) ^b^ | 3.99（2.85-5.22） | 3.73（2.95-5.98） | 19.50±17.20 | 0.538 |
| CREA (μmol/L) ^b^ | 59.60（51.50-67.00） | 61.00（48.00-80.25） | 98.00±31.80 | 0.184 |
| CYS-C (mg/L) ^b^ | 0.98±0.39 | 0.99±0.36 | 2.70±1.85 | 0.647 |
| Uric (umol/L) ^a^ | 283.00±134.00 | 312.00±104.00 | 329.00±0.63 | 0.496 |
| TG (mmol/L) ^b^ | 1.06（0.82-1.43） | 0.94（0.74-1.17） | 1.02±0.25 | 0.222 |
| CHOL (mmol/L) ^a^ | 3.68（3.11-4.46） | 3.95（3.42-4.69） | 4.02±0.73 | 0.140 |
| HDL-C (mmol/L) ^a^ | 2.06（1.62-2.54） | 2.21（1.83-2.73） | 1.55±0.16 | 0.235 |
| LDL-C (mmol/L) ^b^ | 2.25±1.02 | 2.32±0.70 | 2.30±0.97 | 0.159 |
| ALP (IU/L) ^b^ | 104.00±79.50 | 80.40±20.50 | 104..00±1.41 | 0.228 |
| GGT (IU/L) ^b^ | 41.00（24.50-72.00） | 38.00（32.00-88.25） | 45.00±2.82 | 0.200 |

^a^ Data shown as mean ± standard deviation; ^b^data shown as median, interquartile range; ^c^ data shown as number of cases (frequency)
