# Supplementary material for: Genetic and Functional Evaluation of the Role of FOXO1 in Antituberculosis Drug-Induced Hepatotoxicity
Source: Evid Based Complement Alternat Med. 2021 Jun 19;2021:3185874. doi: 10.1155/2021/3185874 (PMC8238576; doi:10.1155/2021/3185874)
Supplement: Supplementary Materials — Figure S1: flow diagram of the study population. Table S1: primer sequences for RT-PCR. Table S2: siRNA sequences targeting FOXO1 used in the study. Table S3: demographic and clinical characteristics and laboratory indicators of enrolled patients. Table S4: candidate single-nucleotide polymorphism of FOXO1 and ALAS1. Table S5: correlation between laboratory indicators and the genotype of the rs2755237 locus. Table S6: correlation between laboratory indicators and the genotype of the rs4435111 locus. Table S7: analysis of the association of genotype distribution and different grades of severity. Table S8: potential biological function annotation for the SNPs related to ATDH. [file 3185874.f1.zip › 3185874.f1/S7 Table. severity.docx]

S7 Table. Analysis of association of genotype distribution and different grade of severity.

| SNP | severity | genotype（n, %）  *p*  22 | | | | | | *p* |
| --- | --- | --- | --- | --- | --- | --- | --- | --- |
|  |  | 11 | | 12 | | 22 | |  |
| rs2755237 | mild | 57 | 68.7% | 25 | 30.1% | 1 | 1.2% | 0.373 |
|  | moderate | 17 | 81.0% | 3 | 14.3% | 1 | 4.8% |  |
|  | severe | 10 | 71.4% | 3 | 21.4% | 1 | 7.1% |  |
| rs4435111 | mild | 59 | 72.0% | 21 | 25.6% | 2 | 2.4% | 0.602 |
|  | moderate | 16 | 76.2% | 5 | 23.8% | 0 | 0.0% |  |
|  | severe | 8 | 57.1% | 6 | 42.9% | 0 | 0.0% |  |
| “11” = mutant homozygote，“12” = heterozygote，“22” = wild homozygote | | | | | | | | |
