# Supplementary material for: Genetic and Functional Evaluation of the Role of FOXO1 in Antituberculosis Drug-Induced Hepatotoxicity
Source: Evid Based Complement Alternat Med. 2021 Jun 19;2021:3185874. doi: 10.1155/2021/3185874 (PMC8238576; doi:10.1155/2021/3185874)
Supplement: Supplementary Materials — Figure S1: flow diagram of the study population. Table S1: primer sequences for RT-PCR. Table S2: siRNA sequences targeting FOXO1 used in the study. Table S3: demographic and clinical characteristics and laboratory indicators of enrolled patients. Table S4: candidate single-nucleotide polymorphism of FOXO1 and ALAS1. Table S5: correlation between laboratory indicators and the genotype of the rs2755237 locus. Table S6: correlation between laboratory indicators and the genotype of the rs4435111 locus. Table S7: analysis of the association of genotype distribution and different grades of severity. Table S8: potential biological function annotation for the SNPs related to ATDH. [file 3185874.f1.zip › 3185874.f1/S8 Table eQTL.docx]

S8 Table. Potential biological function annotation for the SNPs related to ATDH.

|  | ID | Title | PMID | Tissue | Gene | *p* |
| --- | --- | --- | --- | --- | --- | --- |
| rs4435111 | GTEx2015_v6 | The Genotype-Tissue Expression (GTEx) pilot analysis: Multitissue gene regulation in humans | 25954001 | Muscle  Skeletal | WBP4 | 5.66E-08 |
|  | GTEx2015_v6 | The Genotype-Tissue Expression (GTEx) pilot analysis: Multitissue gene regulation in humans | 25954001 | Skin  Sun Exposed  Lower leg | WBP4 | 1.04E-05 |
| rs2755237 | [Vitart V 2010](https://www.ncbi.nlm.nih.gov/pubmed/?term=Vitart%20V%5BAuthor%5D&cauthor=true&cauthor_uid=20719862) | New loci associated with central cornea thickness include COL5A1, AKAP13 and AVGR8 | 20502693 | peripheral blood monocytes | SLC25A19 | 1.15E-07 |
| Data from Genotype-Tissue Expression (GTEx) Project | | | | | | |
