# Supplementary material for: Genetic and Functional Evaluation of the Role of FOXO1 in Antituberculosis Drug-Induced Hepatotoxicity
Source: Evid Based Complement Alternat Med. 2021 Jun 19;2021:3185874. doi: 10.1155/2021/3185874 (PMC8238576; doi:10.1155/2021/3185874)
Supplement: Supplementary Materials — Figure S1: flow diagram of the study population. Table S1: primer sequences for RT-PCR. Table S2: siRNA sequences targeting FOXO1 used in the study. Table S3: demographic and clinical characteristics and laboratory indicators of enrolled patients. Table S4: candidate single-nucleotide polymorphism of FOXO1 and ALAS1. Table S5: correlation between laboratory indicators and the genotype of the rs2755237 locus. Table S6: correlation between laboratory indicators and the genotype of the rs4435111 locus. Table S7: analysis of the association of genotype distribution and different grades of severity. Table S8: potential biological function annotation for the SNPs related to ATDH. [file 3185874.f1.zip › 3185874.f1/S1 Figure.doc]

S1 Figure. Flow diagram of the study population.

**Analysis**

**Enrollment**

Highly suspicious TB patients were consecutively enrolled from December 2016 to April 2018(n=1060)

Excluded (n=287)

♦  without evidenced of tuberculosis (n=243)

♦  with HIV,HBV or HCV (n=16)

♦  with abnormal liver function before treatment (n=28)

Tuberculosis patients with anti-tuberculosis induced liver injury (n=118)

Excluded (n=27)

♦  lost follow up (n= 13)

♦  with use of other hepatotoxic medicine (n=16)

♦  use second-line anti-tuberculosis drugs (n=28)

Receive standard anti-tuberculosis drugs

With good treatment Compliance

(n=746)

Included with definite diagosis of tuberculosis

Six months follow-up treatment and data recording (n=773)

Tuberculosis patients without anti-tuberculosis induced liver injury (n=628)
